# A multivariate Bayesian model for assessing morbidity after coronary artery surgery

**DOI:** 10.1186/cc4951

**Published:** 2006-07-17

**Authors:** Bonizella Biagioli, Sabino Scolletta, Gabriele Cevenini, Emanuela Barbini, Pierpaolo Giomarelli, Paolo Barbini

**Affiliations:** 1 Department of Surgery and Bioengineering, University of Siena, Viale Bracci, 53100 Siena, Italy; 2 Department of Physiopathology, Experimental Medicine and Public Health, University of Siena, Via Aldo Moro, 53100 Siena, Italy

## Abstract

**Introduction:**

Although most risk-stratification scores are derived from preoperative patient variables, there are several intraoperative and postoperative variables that can influence prognosis. Higgins and colleagues previously evaluated the contribution of preoperative, intraoperative and postoperative predictors to the outcome. We developed a Bayes linear model to discriminate morbidity risk after coronary artery bypass grafting and compared it with three different score models: the Higgins' original scoring system, derived from the patient's status on admission to the intensive care unit (ICU), and two models designed and customized to our patient population.

**Methods:**

We analyzed 88 operative risk factors; 1,090 consecutive adult patients who underwent coronary artery bypass grafting were studied. Training and testing data sets of 740 patients and 350 patients, respectively, were used. A stepwise approach enabled selection of an optimal subset of predictor variables. Model discrimination was assessed by receiver operating characteristic (ROC) curves, whereas calibration was measured using the Hosmer-Lemeshow goodness-of-fit test.

**Results:**

A set of 12 preoperative, intraoperative and postoperative predictor variables was identified for the Bayes linear model. Bayes and locally customized score models fitted according to the Hosmer-Lemeshow test. However, the comparison between the areas under the ROC curve proved that the Bayes linear classifier had a significantly higher discrimination capacity than the score models. Calibration and discrimination were both much worse with Higgins' original scoring system.

**Conclusion:**

Most prediction rules use sequential numerical risk scoring to quantify prognosis and are an advanced form of audit. Score models are very attractive tools because their application in routine clinical practice is simple. If locally customized, they also predict patient morbidity in an acceptable manner. The Bayesian model seems to be a feasible alternative. It has better discrimination and can be tailored more easily to individual institutions.

## Introduction

Since the mid-1980s, many predictive models for the assessment of cardiac postoperative mortality have gained popularity in the medical community [1]. Because much has happened in the field of cardiac surgery in recent years, mortality is now low and morbidity has been suggested as both a valid end point and a more attractive target for developing operative risk models [2]. General severity-of-illness models can be inaccurate when applied to specific groups of patients, even if they are valid for comparing outcomes in large numbers of patients [3], and the inaccuracy of these models makes them inappropriate for predicting individual outcome [4,5]. Predictive models, therefore, provide significant advantages in clinical decision-making only if they are customized to the specific population of patients to be investigated. Moreover, although most risk-stratification variables are derived from preoperative patient characteristics [6-10], there are several intraoperative and postoperative physiological variables that can influence morbidity and mortality [11,12].

Higgins and colleagues previously evaluated the relative contribution of preoperative conditions, operating theater events and physiological parameters on admission to the intensive care unit (ICU) to outcome, describing a sequential model derived from the patient's status on admission to the ICU [11]. This model is complementary to the preoperative score of the same study group [13]. Higgins' models, similar to certain other models, use univariate and multivariate logistic regression to quantify prognosis by a numerical scoring system, but caution is needed in applying scores to individuals [14][15][16].

Algorithms for classification derived from the Bayes theorem can be valid alternatives to logistic regression in discrimination problems. The measured set of individual features serves as input to a decision rule by which the patient is assigned to a morbidity risk class. A key characteristic of this approach is that, given complete knowledge of the statistics of the patterns to be classified, the Bayes rule defines the optimum classifier that minimizes the probability of classification error or the expected cost of an incorrect decision [17]. A Bayes linear classifier is the simplest approach, but, in the Bayes sense, it is optimal only for normal distributions with equal covariance matrices of the classification groups. However, in many cases, the simplicity and robustness of the linear classifier compensate for the loss of performance occasioned by nonnormality or nonhomoscedasticity [17-19]. In clinical decision-making it is easy to implement and locally customize, because the statistics of the patterns to be classified only require knowledge of the group means and the pooled within-sample covariance matrix, which can be estimated by a training set of correctly classified cases [18]. The simplicity of a linear classifier, which enables it to be easily tailored and updated to the patient population of a given institution, is a significant advantage of this approach in clinical practice, with respect to multiple logistic regression. The Bayes approach also provides a decision rule for prognosis derived from the whole set of measured predictor variables rather than from scores obtained with logistic regression from group characteristics [20]. These aspects have led to widespread use of the Bayes decision rule in discrimination problems instead of logistic regression [21].

The aims of this study were as follows:

1) to develop an ICU–Bayes model to select the preoperative, intraoperative and postoperative risk factors that best predict postoperative morbidity for coronary artery bypass graft (CABG) patients.

2) to evaluate the reliability of score models in our population of patients.

3) to compare these different models as predictors of morbidity risk.

## Materials and methods

### Patient population

This is an observational study approved by the Ethics Committee of our institution. All patients gave their written, informed consent. All data were entered into a prospectively collected database and retrospectively analyzed for the purposes of this study. The computerized database files of 1,090 consecutive adult CABG patients were analyzed. The database was divided into two subsets: the first consecutive 740 patients, who underwent CABG surgery between 1 January 2002 and 31 December 2003, served as a training set to develop the Bayesian risk model and customize score models to our population of patients; and the next consecutive 350 patients (the testing set), who underwent CABG surgery between 1 January 2004 and 31 December 2004, were used for testing the predictive performance of the models on new data. Standard preoperative and postoperative management and cardiopulmonary bypass (CPB) were performed [22].

### Risk predictor variables included in the model

We selected 88 preoperative, intraoperative and postoperative variables, which could be associated with postoperative morbidity, from the literature. We analyzed the influence of each predictor on outcome. The variable set included all the predictors of both Higgins' models [11,13]. CABG procedures were divided into three periods: pre-CPB, during CPB, and post-CPB. Preoperative and intraoperative data were collected under the anesthesiologist's supervision. Post-CPB consisted of two data-collection periods: data were collected in the first three hours after admission to the ICU, and postoperative outcome data were retrieved from the medical records after discharge from the ICU.

According to the definitions of Higgins and colleagues [11,13], emergency cases were defined as unstable angina, unstable hemodynamics or ischemic valve dysfunction that could not be controlled medically. Left ventricular ejection fractions <35% were considered severely impaired. Diabetes or chronic obstructive pulmonary disease was diagnosed only if the patient was maintained on appropriate medication. CPB time was the total of all bypass runs if a second or subsequent period of bypass was conducted. Re-operation was considered as a separate predictor variable in the analysis [11].

### Outcome variables

The primary outcome in this study was morbidity, which was defined as one or more of the following events:

1) cardiovascular complications: myocardial infarction (documented by electrocardiography and enzyme criteria); low cardiac output requiring inotropic support for >24 hours, an intra-aortic balloon pump (IABP) or a ventricular assist device; or severe arrhythmias requiring treatment or cardiopulmonary resuscitation.

2) respiratory complications: prolonged ventilatory support (defined as mechanical ventilatory support for >24 hours); re-intubation; tracheostomy; clinical evidence of pulmonary embolism or edema; or adult respiratory distress syndrome.

3) neurological complications: central nervous system complications (defined as a focal brain lesion (confirmed by clinical findings or computed tomographic scan, or both), diffuse encephalopathy with >24 hours of severely altered mental status or unexplained failure to awaken within 24 hours of the operation).

4) renal complications: acute renal failure (need for dialysis).

5) infectious complications: serious infection was defined as culture-proven pneumonia, mediastinitis, wound infection, septicemia (with appropriate clinical findings) or septic shock.

6) hemorrhagic complications: bleeding requiring re-operation.

Different authors tend to use their own criteria to compare the performances of different risk models for predicting morbidity [23-25]. We preferred the outcome criteria for morbidity in the original database of the Cleveland Clinic Foundation, from which the scoring systems were derived [11,13], to evaluate the reliability of Higgins' scores to predict complications in our patient population.

### Bayes linear model

For discriminating patients at risk of morbidity (M) from those with a normal clinical course (N; or at low risk of morbidity) a Bayes classification scheme was used [20,25,26]. Using the set of measured variables (**x**) for a patient, the Bayes rule enables morbidity risk evaluation directly through the posterior conditional probability of morbidity:



(Where *P*(M) is the prior probability of morbidity, *P*(N) = 1 - *P*(M) is the prior probability of normal course, and *p*(**x**|M) and *p*(**x**|N) are the conditional probability density functions (CPDFs) of morbid patients and of normally recovering patients, respectively.)

Similarly, the posterior conditional probability of normal course is as follows:



A reasonable discrimination criterion would be to assign patient **x **to the population with the largest posterior probability, but the decision rule can also be chosen using somewhat different reasoning [17].

If no assumptions at all are made about the form of the CPDFs, these functions must be estimated from the training set (correctly classified cases for both classes of patient) by certain nonparametric methods [18]. Despite recent interest in these nonparametric methods, the overwhelming majority of applications of discrimination and classification still rely on various parametric assumptions. In this paper, we assumed normal CPDFs with equal covariance matrices, because in many cases this choice provides a simple and robust method of discrimination, especially when many variables are available and have to be selected [17-19]. The practical benefits of making this assumption are that the discriminant function and allocation rule become very simple indeed. In particular, according to these hypotheses, the decision boundary for discrimination is given by a linear function in **x **and the corresponding model is, therefore, described as linear [17]. In addition, the CPDFs are easily estimated and locally tuned, because they require only the calculation of group means and the pooled within-sample covariance matrix. Indeed, the CPDF of group i (i = M or N) is given by the well-known multivariate normal probability density as follows:



(Where **μ**_i _is the mean of class i, **∑ **is the covariance matrix (which is assumed to be the same for M and N), q is the number of predictor variables used for discrimination and the superscript T indicates matrix transposition.)

Of course, in our model **μ**_M_, **μ**_N _and **∑ **were estimated from the means and covariance matrix, which were calculated from the training set of patients in classes M and N. The prior probabilities *P*(M) and *P*(N) were both assumed to be 0.5.

A stepwise approach was used to select an optimal subset of predictor variables to be included in the Bayesian model [3,25,26]. The capacity of the model to discriminate between patients who will have complications after surgery and patients who will have a normal clinical course was assessed from the receiver operating characteristic (ROC) curves [16]. The goodness of fit of the Bayesian model was evaluated using the Hosmer-Lemeshow χ^2 ^statistic [15]. Finally, testing data were used to evaluate the model's generalization capacity. All computer calculations for the Bayesian model were performed using the MATLAB^® ^software package (The MathWorks Inc., Natick, MA, USA) [27].

### ROC curves

It is well known that ROC curves give a graphic representation of the relationship between the true-positive fraction (TPF) and the false-positive fraction (FPF). ROC curves can be used to study the effect of changing the discrimination criterion, namely of selecting a probability threshold to be compared with the predicted model probability of morbidity [28]. By using the sensitivity (SE) and specificity (SP) values, the ROC curve is obtained by plotting SE = TPF against 1 - SP = FPF in a squared box, where the area under the ROC curve (AUC) is commonly used to measure the predictive power of the statistical discrimination model [28-30].

The discrimination criterion assumed in our model was settled by choosing the point on the ROC curve where SE = SP. If the two classes of patients have equal prior probabilities and normal distributions with equal covariance matrices, this choice provides an optimal discrimination rule, minimizing the probability of error [17]. The corresponding decision probability was taken as the threshold to discriminate between high and low risk of morbidity. Of course, different criteria (such as, different pairs of SE and SP) can be chosen, depending on the clinical cost of a wrong decision [17].

The discrimination performance of the model was evaluated by analyzing the ROC curve and its 95% confidence interval, which was fitted from the training set using a maximum-likelihood estimation procedure by assuming binormal distribution of the data [30].

### Model generalization

A key element in statistical discrimination is the model's generalization capacity, which is estimated by the model's performance on a test set that is not used for training. The model generalizes well if errors in testing and training sets do not differ significantly. A well-known source of loss of generalization power is the use of too many predictor variables [31]. A greater number (q) of predictor variables requires a greater number of parameters (q for each group mean and  for the pooled within-sample covariance matrix) to be estimated to define normal CPDFs. Of course, with a set of training data, the accuracy of the model's parameter estimates rapidly worsens as q increases, leading to a significant loss in generalization capacity. A minimum subset of predictor variables (also called 'features') that provides high generalization power to the Bayes linear classifier should, therefore, be sought using an optimization criterion. We used a computer-aided stepwise technique [32] combined with the leave-one-out (LOO) method of cross-validation [33] to check the model generalization directly during the feature-selection process. At each step of the process, a variable was entered or removed from the predictor subset and the significance of its contribution to the AUC was evaluated. The stepwise process stopped if no variable satisfied the criterion for inclusion or removal. The LOO method is particularly useful in biomedical applications where little data is usually available, because it enables all the data to be used efficiently for training the classification model and testing its predictive performance. For *n *available input–output data, it considers *n *distinct training sessions combining (*n *- 1) cases in all possible ways. The *n *cases left out, one per session, were used to calculate the testing discrimination performance, which was evaluated by the AUC.

### Comparison of score models with the Bayesian model

Because comparison of a locally customized model with a previously published model can be unfair, the method proposed by Higgins and colleagues for assessing morbidity risk in the ICU [11] was employed to design score models customized to our patient sample. The following two different approaches were used:

1) exactly following Higgins' procedure, including the selection of variables.

2) tailoring a score model with the variables selected for the Bayesian model to our data set.

The first choice, the fully customized (FC) score model, enables comparison of our proposed Bayes model with the best-possible score model designed from our patient sample by mimicking Higgins' method. The second choice, the partially customized (PC) score model, was built to evaluate differences in model performance when the same predictor variables were used.

Bayesian and score models were compared for discrimination and calibration [29]. Model discrimination was tested by analyzing the ROC curves derived from the technique developed by Metz *et al*. [30]. Model calibration was evaluated using the Hosmer-Lemeshow goodness-of-fit test [15].

All computer calculations for score models were performed using the SPSS^® ^statistical package (SPSS Inc., Chicago, IL, USA) [34].

## Results

### Demographics, morbidity and mortality rate

Demographics, baseline patient characteristics, main operative data and morbidity outcomes for both training (740 patients) and testing (350 patients) sets are shown in Table 1. In-ICU morbidity was around 21%, whereas a mortality rate of about 2% was recorded within 30 days of the operation. This small mortality group was regarded as unsuitable for validating the score risk models and performing a Bayes analysis with respect to the mortality end point.

### Bayes linear model

The stepwise procedure selected 12 variables (Table 2). Univariate analysis indicated that the oxygen delivery index (DO_2_I) was the most discriminating variable and it was, therefore, selected as the first step of the stepwise procedure. In the following steps, addition of other variables produced increases in the AUC, which reached a value about 13% greater at the final step than at the first step (Table 2). After calibrating the Bayesian model with this set of predictor variables, the Hosmer-Lemeshow test showed a good fit (*P *= 0.35).

**Figure 1:**
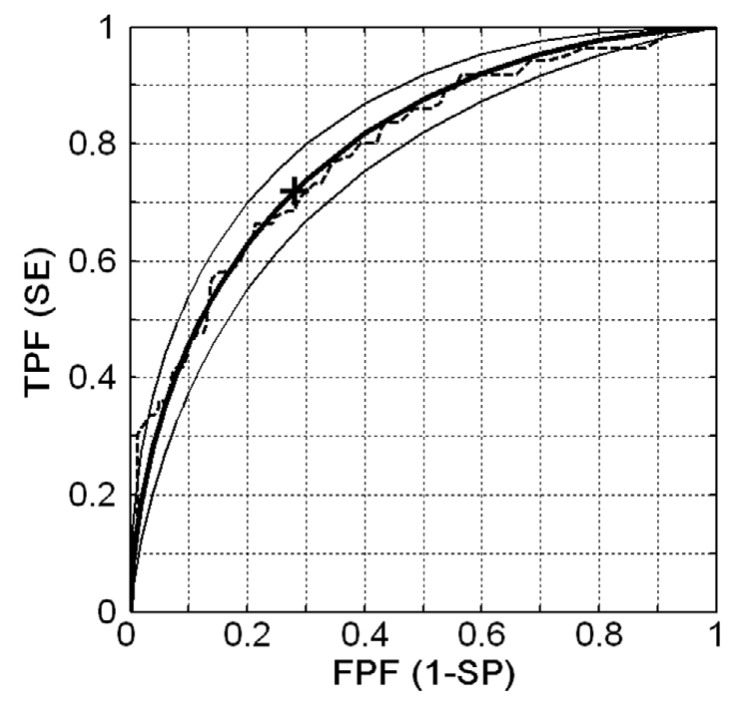
Receiver operating characteristic (ROC) curve for the Bayes linear model. The ROC curve (bold continuous line) and its 95% confidence interval (fine continuous lines) were derived from the training set by a maximum-likelihood estimation procedure by assuming binormal distribution of the data. The corresponding area under the ROC curve was 0.80 (with a 95% confidence interval ranging from 0.75 to 0.83). The cross indicates the point where SE and SP are equal. The dashed line represents the empirical ROC curve obtained from testing data. FPF, false-positive fraction; SE, sensitivity; SP, specificity; TPF, true-positive fraction.

Figure 1 shows the ROC curve (bold line) and its 95% confidence interval (fine lines), which were estimated by the training set by assuming binormal distribution of the data. The corresponding AUC was 0.80 (with a 95% confidence interval ranging from 0.75 to 0.83). The empirical ROC curve obtained from the testing set is the dashed line in the same figure and the corresponding AUC is equal to 0.79. The ROC curve and AUC for the testing set are very close to the training set estimates, indicating that the Bayes linear model designed from training set maintains very similar discrimination performance with new data.

The cross on the estimated ROC curve indicates the point at which SE = SP (72%). This choice corresponded to a probability threshold of 0.427: patients with an estimated posterior probability of morbidity greater than or equal to 0.427 were classified as at high risk. With this decision criterion, the percentage of correctly predicted cases in the testing set was 70.6% (247 out of 350 patients): the Bayesian model correctly recognized 61 out of 86 morbidity cases (70.9%) and 186 out of 264 uncomplicated cases (70.5%). This value is within the confidence interval of the ROC curve estimated with the training data (Figure 1).

More specifically, all patients in the testing set who developed infections were correctly identified by the model as at high risk. The performance of the prediction model deteriorated slightly if patients had other types of complications (91%, 79%, 77%, 74% and 62% for renal, cardiovascular, respiratory, neurological and hemorrhagic complications, respectively). The high percentages obtained for most complications are not surprising because high-risk patients often had matching complications. In fact, algorithm performance improved sharply as the number of concomitant complications increased. Again, in the test set 61%, 79%, 89% and 100% of morbidity cases were correctly discriminated when the number of complications was one, two, three or more than three, respectively.

### Fully customized score model

**Figure 2:**
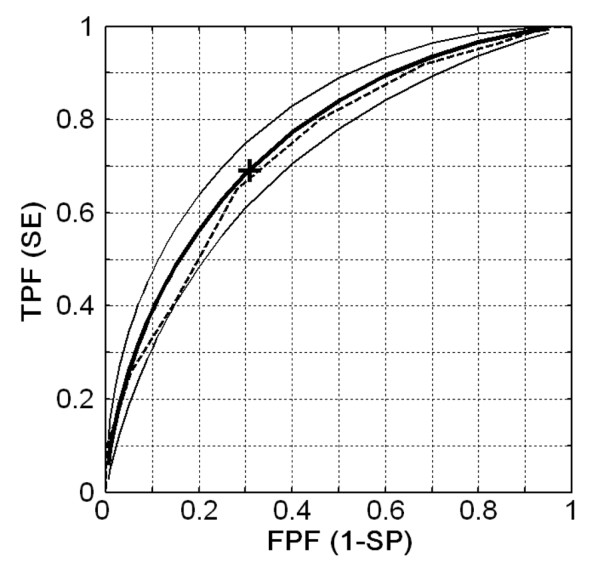
Receiver operating characteristic (ROC) curve for the fully-customized score model. The ROC curve (bold continuous line) and its 95% confidence interval (fine continuous lines) were derived from the training set by a maximum-likelihood estimation procedure by assuming binormal distribution of the data. The corresponding area under the ROC curve was 0.76 (with a 95% confidence interval ranging from 0.72 to 0.80). The cross indicates the point where SE and SP are equal. The dashed line represents the empirical ROC curve obtained from testing data. FPF, false-positive fraction; SE, sensitivity; SP, specificity; TPF, true-positive fraction.

Locally selected variables and corresponding scores of the FC model are shown in Table 3. The Hosmer-Lemeshow test proved that the FC score model fits our data well. Regarding the discrimination capacity, Figure 2 shows both the estimation of the ROC curve (bold line) derived from the training set using the binormal method and the empirical ROC curve (dashed line) obtained with the testing set. The 95% confidence interval of the estimated ROC curve is bounded by the two fine lines in the same figure. The AUC corresponding to the estimated ROC curve was 0.76 (with a 95% confidence interval ranging from 0.72 to 0.80), whereas the AUC calculated from testing data was 0.74. Also for this model, the discrimination performance was similar in testing data and training set estimates. As for the Bayesian model, we assumed a decision criterion, such as to obtain the most similar possible values of SE and SP. This corresponded to a threshold score of 4 (that is to say, patients with a score greater than or equal to 4 were classified at high risk of morbidity).

The FC score model seems to have lower discrimination capacity than the Bayes linear classifier, because the AUC obtained from the former model was lower than the AUC corresponding to the latter model. The Metz technique for comparison of ROC areas proved a significant difference between the AUCs of the two models (*P *< 0.05).

### Partially customized score model

Table 4 shows the scores associated with the PC score model: according to the model construction criterion, the variables in the PC score model were the same as those in the Bayes model. The Hosmer-Lemeshow goodness-of-fit test demonstrated good calibration.

**Figure 3:**
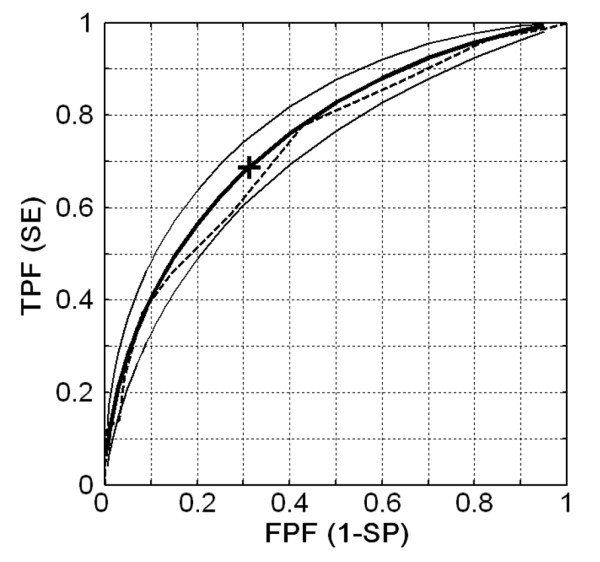
Receiver operating characteristic (ROC) curve for the partially-customized score model. The ROC curve (bold continuous line) and its 95% confidence interval (fine continuous lines) were derived from the training set by a maximum-likelihood estimation procedure by assuming binormal distribution of the data. The corresponding area under the ROC curve was 0.75 (with a 95% confidence interval ranging from 0.71 to 0.80). The cross indicates the point where SE and SP are equal. The dashed line represents the empirical ROC curve obtained from testing data. FPF, false-positive fraction; SE, sensitivity; SP, specificity; TPF, true-positive fraction.

Figure 3 shows the estimation of the ROC curve and its 95% confidence interval that were obtained from the training set, by assuming binormal distribution of the data, and the empirical ROC curve derived from the testing set. The AUC of the estimated ROC curve was 0.75 (with a 95% confidence interval ranging from 0.71 to 0.80), whereas the AUC appraised from the testing data was 0.73. The discrimination capacity of the PC model was, therefore, clearly worse than that of the Bayesian model: the Metz test for ROC area comparison indicated high statistical significance (*P *< 0.01). The threshold score, which corresponded to about equal SE and SP, was the same as for the FC score model (that is exactly = 4).

### Higgins' score model

**Figure 4:**
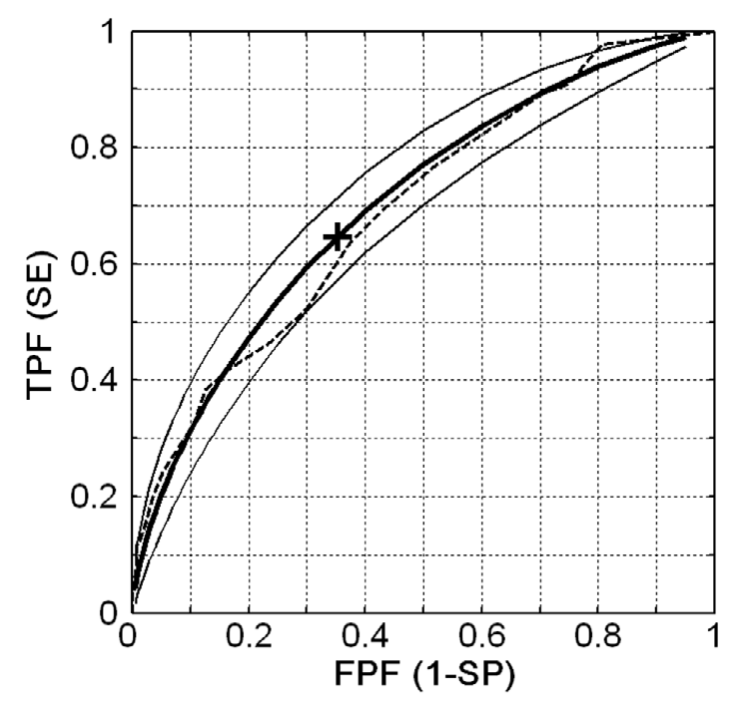
Receiver operating characteristic (ROC) curve for the standard (uncustomized) Higgins score model. The ROC curve (bold continuous line) and its 95% confidence interval (fine continuous lines) were derived from the training set by a maximum-likelihood estimation procedure by assuming binormal distribution of the data. The corresponding area under the ROC curve was 0.70 (with a 95% confidence interval ranging from 0.65 to 0.75). The cross indicates the point where SE and SP are equal. The dashed line represents the empirical ROC curve obtained from testing data. FPF, false-positive fraction; SE, sensitivity; SP, specificity; TPF, true-positive fraction.

The performance of the score model became radically worse when morbidity risk in our ICU patients was discriminated using the standard scoring system, as originally proposed by Higgins *et al*. [11]. This is evident comparing Figures 1, 2, 3 with Figure 4, which shows the ROC curves obtained for the Higgins' original scoring system with preoperative conditions, operating theater events and measurements on admission to the ICU. In this case, AUC values were 0.70 and 0.69 when calculated by estimated and empirical ROC curves, respectively. A score threshold of 6 was chosen to obtain approximately equal SE and SP.

### Bayesian model application

An example of the application of the Bayesian model is shown in Table 5. Two patients were classified, one who did not develop complications (case A) and one who only developed a cardiovascular complication (case B). Table 5 shows the values of the 12 predictor variables (**x**) for both cases and those of the CPDFs for morbidity and normal conditions (*p*(**x**|M) and *p*(**x**|N), respectively). *p*(**x**|M) and *p*(**x**|N) were calculated using equation 3 (above), which required knowledge of the group means and covariance matrix (calculated from the training set) and involved the use of a personal computer. Assuming *P*(M) = *P*(N) = 0.5, the Bayes posterior probability of morbidity was derived from equation 1 (above). Because this posterior probability was less than the selected probability threshold (0.427) in case A, whereas in case B it was greater, both patients were correctly classified. By contrast, cases A and B were both classified as high risk by all score models (Table 5).

## Discussion

A model that predicts the outcome of patients in the ICU with good discrimination can be useful because the risk prediction enables better allocation of resources, for example, and can aid decisions about the appropriateness of continuing treatment [35]. Most studies have concentrated on short-term mortality, and there is a lack of easy-to-use models predicting risk of complications (morbidity). However, mortality by itself might not be an adequate indicator of quality of care or resource use [2,14]. On the contrary, morbidity might be more informative, being a more frequent event than mortality and enabling statistical inferences to be drawn from smaller populations. Finally, morbidity can be measured in terms of postoperative complications and length of stay in the ICU [1,14]. Several authors have developed predictive indices of stay in the ICU after heart surgery. Most of these studies included preoperative variables and, generally, did not take events affecting patient outcome in the operative or immediate postoperative period into account. Of course, quantifying risk and assessing outcome in the ICU after cardiac surgery according to preoperative variables alone could lead to incorrect conclusions about the true morbidity risk [1,11,14]. We chose to consider the contribution of preoperative conditions, operating theater events and physiological measurements on admission to the ICU and selected an optimal subset of predictor variables using a stepwise technique. The aim of the study was to compare two approaches for risk discrimination in ICU patients after heart surgery: a Bayes linear classifier developed in our specialized ICU, and score models designed in our training set using the method proposed by Higgins and colleagues [11].

Both approaches have strengths and weaknesses. The greatest benefit of a score model is that it only requires the sum of integer factors and is, therefore, very simple to apply in routine clinical practice. However, the Higgins approach first requires the development of a logistic regression model. Although continuous and categorical predictor variables can be mixed, the model development can be problematic because logistic regression is very sensitive to correlations between predictors in the model [16]. If the predictor variables are highly correlated during local application, the result is a loss of information. To overcome this problem, we used a stepwise procedure, similar to that employed with the Bayes model, to select variables to enter in the logistic regression model. A weakness of the scoring system is the difficulty of locally customizing this type of model if training sets planned in a different institution are used. The design of the scoring system requires a complex process, which can have low interobserver reproducibility. In particular, to refit the logistic model using all predictors as categorical variables, Higgins and colleagues used a locally weighted smoothing scatterplot procedure, which involves subjective choices, to identify cut-off points. Similar difficulties might also be encountered when the model is updated with new data, such as improved results resulting from technological advances. Easy updating is a crucial feature. In fact, acquisition of correctly classified new patients enables the training set to be increased day by day, with corresponding improvement in discrimination performance of the model. Progress in medical techniques also makes it necessary to be able to change decision-making models continuously. For example, the dramatic decrease in cardiac postoperative mortality means that morbidity is now used as the new end point for developing operative risk models. Bayes linear discrimination provides much more ductile models because their tuning to new data sets is a rapid and objective procedure that only requires calculation of predictor variable means in the two risk classes and pooled variances and covariances in the whole training set. A weakness of this approach is that it is optimal only if the CPDFs of the two classes can be assumed normal and with equal covariance matrices. However, this type of classifier is used in a wide range of clinical applications because its simplicity and robustness compensate for the loss of performance resulting from incomplete observance of the above statistical hypotheses [17-19]. Our results show that the Bayes linear classifier can predict all types of complications, especially infection and renal failure. Discrimination increases with the number of complications. In particular, the model exactly recognized patients with more than three complications.

The area under the ROC curve, estimated by a maximum-likelihood procedure by assuming binormal distribution of the data, was significantly higher for the Bayes linear model. Similar results were obtained by evaluating the empirical ROC curves obtained from the testing set. According to the Hosmer and Lemeshow criterion [15], all locally customized models had acceptable discrimination capacities in the testing data set, because their AUCs were much greater than 0.7 and less than 0.8. On the contrary, the AUC of Higgins' standard scoring system calculated with the testing data set did not reach 0.7, indicating poor discrimination capacity for this model in our patients. With regard to calibration, the Hosmer-Lemeshow test showed good fit for all models, except Higgins' scoring system. Table 5 sums up the discrimination and calibration performances tested for the Bayes linear classifier and FC, PC and Higgins' standard scoring models. It points out that the two locally customized score models had significantly lower discrimination capacities than the Bayes linear classifier. The statistical significance of the difference in AUCs between the Bayes linear classifier and the score models increased when passing from the FC to the PC approach, indicating that the score model performance considerably worsened when using the set of variables identified as optimal by the Bayes classifier as predictors. Furthermore, Table 5 shows the weak points of the Higgins' standard score system applied in our specialized ICU, confirming that any comparison of a locally customized model with a previously published model is unfair, regardless of the method by which the model was developed.

In our data set, model performance dropped sharply when logistic regression models were changed to scoring systems, using the procedure suggested by Higgins *et al*. [11]. In fact, when we customized logistic models without transforming regression coefficients into integer scores, we obtained discrimination performance only slightly worse than that of the Bayesian model; however, in this case statistical comparison of ROC areas did not indicate significant differences. This fully agrees with the results obtained in previous studies [21,36] and suggests that attempts to obtain a very simple clinical model that reduces computation difficulties could lead to significant loss of performance. Despite the immediateness and simplicity of scoring systems derived from weighted variables, sequential summing of integer factors can distort the multivariate characteristics of outcome prediction. The Bayesian model does not use a weighted scoring system, it uses a decision rule that enables the probability of morbidity in patients undergone CABG surgery to be assessed according to multivariate statistics of the predictor variables used for discrimination (12 variables were selected in our model).

Many papers have tested the validity of the preoperative scoring system [37-42], but to our knowledge, no study on validation of Higgins' ICU-admission score has been published. The present study is the first to locally customize this ICU scoring system and to test its validity using external data. In the original version of the ICU-admission morbidity model, Higgins and associates used an additive scoring system comprising 13 weighted predictors that were graded from 1 to 7, giving a maximum total score of 44 points [11]. In the FC version, the same method of model development led to a different choice of 13 weighted predictors. Most risk predictors in Higgins' score are the same in other North American and European mortality risk models (such as; the Parsonnet and EUROscore models) [43-45]. Similar risk factors were revealed by Higgins and colleagues and in our Bayes model (Table 2): emergency procedure, age, elevated serum creatinine levels, prior heart operation, history of vascular disease, weight, CPB time, use of IABP after CPB, and low postoperative flow state (low cardiac index and low DO_2_I). Although a low preoperative ejection fraction is a known predictor of poor immediate postoperative outcome after cardiac surgery, it was not a risk factor in our study. This is in line with the findings of Zaroff and colleagues [46], who showed that in some high-risk cases there could be great improvement in left ventricular function after operation because of successful revascularization. Not all patients with a low preoperative ejection fraction required inotropic support, and a low ejection fraction was not a risk factor for outcome for the whole population [46]. However, we found that morbidity was associated with the need for preoperative and postoperative IABP and use of inotropes after the operation, and these variables are strongly correlated to poor cardiac function.

The idea of developing a risk model derived from the Bayes rule is not new. In 1985, Edwards and colleagues began to use a Bayesian model of operative mortality associated with CABG procedures [20]. The Society of Thoracic Surgeons National Cardiac Surgery Database model, developed by Edwards and colleagues, incorporates 23 risk factors and is the most widely used model in the USA [47]. The Society of Cardiothoracic Surgeons of Great Britain and Ireland also proposed a Bayesian model for CABG patients in the UK [48,49]. However, both these models focused on postoperative mortality, not morbidity. In the present study, we developed and tested a Bayesian discrimination model for assessing morbidity risk after coronary artery surgery. Some practical aspects need to be considered when this discrimination technique is chosen as support for clinical decision-making. First of all, this approach requires the use of a computer. Moreover, an initial retrospective study for deriving the model might be time consuming and tedious. If detailed records are not available, it might not be possible to obtain the whole set of variables for each patient. Finally, many groups have found it necessary to establish physician training programs to ensure that all users of the model have the same interpretation of terminology and results [20,25,26].

Conclusive remarks must also be made about possible limits of this study. Firstly, the model was developed and validated in a single institution with a relatively low surgical volume, and it might not reflect the experience of hospitals performing a different number of CABG operations, because outcomes can be related to surgical volume [1,14,50]. Moreover, patients were treated by a small, experienced team, and this decreased the variability resulting from perioperative factors. Secondly, we created a multivariate model with 12 predictor variables, not a simple risk score. Not all physicians will find it easy to use, because it requires a special software program to estimate the risk of morbidity. However, our results show that transforming complex statistical models into simple score systems might lead to a significant loss of discrimination performance. On the other hand, personal computers are widely used for managing patient data in ICUs, so introduction of software for estimating the risk of morbidity would not be unduly onerous. Thirdly, the model is derived from preoperative, intraoperative and postoperative variables and only allows prediction of morbidity after ICU admission. Because the model does not assess risk solely on the patient's preoperative status, it cannot be used to enhance patient counseling. Another preoperative risk model needs to be used to define the risk and planning of surgical procedures and type of anesthesia before the operation. Finally, because the duration of CPB was an intraoperative risk factor for morbidity in our model, we might expect the risk of morbidity to be incorrectly estimated in off-pump patients using this risk model.

## Conclusion

In this paper, we compared two approaches for morbidity risk discrimination of ICU patients after heart surgery: a Bayes linear classifier developed in our specialized ICU, and score models customized to our data set. A significant advantage of score models is their simplicity, which stems from the use of integer scores, making them readily applied in routine clinical practice. However, our results showed that transforming multivariate statistical models into simplified score systems can lead to a significant loss of discrimination performance. A weakness is the difficulty of locally customizing score models for individual institutions. The Bayes linear classifier is much easier to customize to individual institutions and update with new sets of data, but it requires a special software program to estimate the risk of morbidity. Regarding the discrimination capacity of the two approaches, the results suggest that Bayes classifiers perform significantly better than scoring systems using the same predictor variables. This does not mean that Bayesian models are always the best, especially if it is appropriate to reduce computational difficulties. However, knowledge of the weaknesses and strengths of both methods makes rational choice possible, guiding clinical users towards the more convenient model for assessing morbidity in the ICU after coronary artery surgery.

## Key messages

• 		Bayes linear classifiers and score models for predicting morbidity risk in intensive care patients after coronary artery surgery are compared.

• 		A set of few clinical predictors was chosen for both models using a stepwise selection procedure.

• 		Discrimination capacity, measured by comparing the area under the ROC curves, was significantly better in the Bayesian model.

• 		The score models were simple to apply, largely because of the limited computation involved, but were associated with a significant loss of predictive power.

• 		Any comparison between different approaches for morbidity risk discrimination is fair only if all models are locally customized.

## Abbreviations

AUC = area under the ROC curve; CABG = coronary artery bypass graft; CPB = cardiopulmonary bypass; CPDF = conditional probability density function; DO_2_I = oxygen delivery index; FC = fully customized; FPF = false-positive fraction; IABP = intra-aortic balloon pump; ICU = intensive care unit; LOO = leave one out; PC = partially customized; ROC = receiver operating characteristic; SE = sensitivity; SP = specificity; TPF = true-positive fraction.

## Competing interests

The authors declare that they have no competing interests.

## Authors' contributions

All authors participated in study planning and co-ordination. SS collected clinical data. SS, BB and PG were concerned with all clinical aspects of the study. GC, EB and PB designed the Bayesian classifier and locally customized score models and performed the statistical analysis. All authors read and approved the final manuscript.

## Figures and Tables

**Table 1:** Demographics, baseline patient characteristics, main operative data and morbidity outcomes

**Variable**	**Training data**		**Testing data**	
	**Mean or N**	**SD or %**	**Mean or N**	**SD or %**
Age (years)	67.3	8.7	67.9	9.3
Gender (female)	195	26%	79	23%
Weight (kg)	72.7	11.6	72.3	10.9
BSA (m^2^)	1.80	0.17	1.79	0.16
Preoperative Hct (%)	38.5	4.8	39.8	4.6
Preoperative creatinine (mg/dL)	1.07	0.49	1.10	0.36
Albumin (g/dL)	3.80	0.43	3.98	0.39
Treated COPD	73	9.9%	26	7.4%
Preoperative arrhythmia	98	13%	60	17%
CHF	43	5.8%	12	3.4%
History of PVD/CD	137	19%	83	24%
History of TIA, stroke	28	3.8%	27	7.7%
Preoperative IABP	21	2.8%	6	1.7%
MI ≤30 days	163	22%	75	21%
Treated diabetes	144	19%	54	15%
LVEF ≤35%	64	8.6%	35	10%
Emergency operation	21	2.8%	10	2.9%
Urgent operation	71	9.6%	22	6.3%
REDO	19	2.6%	10	2.9%
Duration of CPB (minutes)	112	45	118	46
Aortic cross-clamp time (minutes)	79	33	81	33
Cardiovascular complications	81	11%	53	15%
Respiratory complications	46	6.2%	26	7.4%
Neurological complications	28	3.8%	19	5.4%
Renal complications	22	3.0%	11	3.1%
Infectious complications	8	1.1%	7	2.0%
Hemorrhagic complications	33	4.5%	21	6.0%

BSA, body surface area; CHF, congestive heart failure; COPD, chronic obstructive pulmonary disease; CPB, cardiopulmonary bypass; Hct, hematocrit; IABP, intra-aortic balloon pump; LVEF, left ventricular ejection fraction; MI, myocardial infarction; N, number of cases; PVD/CD, peripheral vascular disease/carotid disease; REDO, re-operation; SD, standard deviation; TIA, transient ischemic attack.

**Table 2:** Stepwise selection of variables for discriminating morbidity with the Bayesian model

**STEP**	**Variable**	**Area under ROC curve**
1	DO_2_I (mL/minute/m^2^) after 3 hours in ICU	0.7080
2	Inotropic support after CPB	0.7407
3	PVD and/or CD	0.7573
4	Preoperative creatinine (mg/dL)	0.7692
5	IABP after CABG	0.7771
6	Weight (kg)	0.7827
7	REDO	0.7876
8	Duration of CPB (minutes)	0.7923
9	Age (years)	0.7949
10	WBC (10^3^/mm^3^) after 3 hours in ICU	0.7966
11	Preoperative IABP	0.7976
12	Emergency operation	0.7988

The area under the receiver operating characteristic (ROC) curve represents discrimination performance. The stepwise technique was employed in combination with the leave-one-out method of cross-validation. The variable that adds the most or least to the area under the ROC curve is entered or removed, respectively, at each step. The stepwise process stopped when no further significant improvement was found. CABG, coronary artery bypass graft; CD, carotid disease; CPB, cardiopulmonary bypass; DO_2_I, oxygen delivery index; IABP, intra-aortic balloon pump; ICU, intensive care unit; PVD, peripheral vascular disease; REDO, re-operation; WBC, white blood cells.

**Table 3:** Fully customized score model

**Variable**	**RC**	**SE**	**OR**	**95% CI**	**Score**
PAH	1.40	0.61	4.04	1.23–13.2	3
PVD and/or CD	0.91	0.24	2.49	1.56–3.98	2
Treated diabetes	0.57	0.25	1.77	1.09–2.88	1
LVEF ≤35%	0.32	0.36	1.37	0.68–2.76	1
IABP after CABG	2.93	0.85	18.7	3.52–99.2	6
Inotropic support after CPB	0.96	0.24	2.61	1.64–4.16	2
Age ≥70 years	0.34	0.22	1.40	0.91–2.16	1
Preoperative creatinine ≥1.2 mg/dL	0.24	0.22	1.27	0.83–1.95	1
Duration of CPB ≥2 hours	0.27	0.22	1.31	0.86–2.01	1
T ≤35°C	0.49	0.23	1.64	1.05–2.56	1
SvO_2_≤62.5%	0.53	0.26	1.69	1.03–2.79	1
VCO_2_≤175 mL/minute	0.37	0.26	1.45	0.86–2.42	1
DO_2_I ≤300 mL/minute/m^2^	0.71	0.30	2.04	1.15–3.62	1
Constant	-5.11	1.18			

CABG, coronary artery bypass graft; CD, carotid disease; CI, confidence interval; CPB, cardiopulmonary bypass; DO_2_I, oxygen delivery index; IABP, intra-aortic balloon pump; LVEF, left ventricle ejection fraction; OR, odds ratio; PAH, pulmonary artery hypertension; PVD, peripheral vascular disease; RC, regression coefficient; SE, standard error; SvO_2_, mixed venous oxygen saturation; T, body temperature; VCO_2_, carbonic dioxide production.

**Table 4:** Partially customized score model

**Variable**	**RC**	**SE**	**OR**	**95% CI**	**Score**
DO_2_I ≤300 mL/minute/m^2^	1.19	0.22	3.27	2.13–5.02	2
Inotropic support after CPB	1.07	0.28	2.91	1.68–5.06	2
PVD and/or CD	0.92	0.24	2.51	1.58–4.01	2
Preoperative creatinine ≥1.2 mg/dL	0.36	0.23	1.43	0.91–2.24	1
IABP after CABG	3.48	1.11	32.6	3.68–288	7
Weight ≤72 kg	0.24	0.22	1.27	0.83–1.96	1
REDO	3.15	1.77	23.3	0.73–745	6
Duration of CPB ≥2 hours	0.53	0.21	1.70	1.12–2.58	1
Age ≥70 years	0.44	0.22	1.55	1.01–2.38	1
WBC ≥12000/mm^3^	0.21	0.21	1.24	0.81–1.88	1
Preoperative IABP	1.14	0.91	3.13	0.52–18.8	2
Emergency operation	0.50	0.33	1.65	0.86–3.18	1
Constant	-5.65	1.22			

CABG, coronary artery bypass graft; CD, carotid disease; CI, confidence interval; CPB, cardiopulmonary bypass; DO_2_I, oxygen delivery index; IABP, intra-aortic balloon pump; OR, odds ratio; PVD, peripheral vascular disease; RC, regression coefficient; REDO, re-operation; SE, standard error; WBC, white blood cells.

**Table 5:** Examples of application of the Bayesian model

**Variables and model calculations**	**Case A**	**Case B**
DO_2_I (mL/minute/m^2^)	427.9	332.3
Inotropic support after CPB	NO	NO
PVD and/or CD	YES	NO
Preoperative creatinine (mg/dL)	1.0	1.2
IABP after CABG	NO	NO
Weight (kg)	74	52
REDO	NO	NO
Duration of CPB (minutes)	150	185
Age (years)	53	73
WBC (10^3^/mm^3^)	12	7.97
Preoperative IABP	NO	NO
Emergency operation	NO	NO
*p*(**x**|N)	1.24 × 10^-11^	4.29 × 10^-11^
*p*(**x**|M)	7.03 × 10^-12^	4.80 × 10^-11^
Bayes posterior probability of morbidity (DT = 0.427)	0.361 (LoRi)	0.528 (HiRi)
FC score (DT = 4)	5 (HiRi)	6 (HiRi)
PC score (DT = 4)	4 (HiRi)	4 (HiRi)
Higgins score (DT = 6)	10 (HiRi)	10 (HiRi)

CABG, coronary artery bypass graft; Case A, a patient without complications; Case B, a patient with a cardiovascular complication; CD, carotid disease; CPB, cardiopulmonary bypass; DO_2_I, oxygen delivery index; DT, decision threshold; FC, fully customized; HiRi, classification at high risk of morbidity; IABP, intra-aortic balloon pump; LoRi, classification at low risk of morbidity; PVD, peripheral vascular disease; PC, partially customized; *p*(**x**|M), probability density function of morbidity; *p*(**x**|N), probability density function of normal condition; REDO, re-operation; WBC, white blood cells.
